# Polymorphisms in Nucleotide Excision Repair Genes, Arsenic Exposure, and Non-Melanoma Skin Cancer in New Hampshire

**DOI:** 10.1289/ehp.10096

**Published:** 2007-06-11

**Authors:** Katie M. Applebaum, Margaret R. Karagas, David J. Hunter, Paul J. Catalano, Steven H. Byler, Steve Morris, Heather H. Nelson

**Affiliations:** 1 Department of Epidemiology and; 2 Department of Environmental Health, Harvard School of Public Health, Boston, Massachusetts, USA; 3 Section of Biostatistics and Epidemiology, Department of Community and Family Medicine, and the Norris Cotton Cancer Center, Dartmouth Medical School, Lebanon, New Hampshire, USA; 4 Channing Laboratory, Department of Medicine, Brigham and Women’s Hospital and Harvard Medical School, Boston, Massachusetts, USA; 5 Department of Biostatistics, Harvard School of Public Health, Boston, Massachusetts, USA; 6 Department of Biostatistical Science, Dana Farber Cancer Institute, Boston, Massachusetts, USA; 7 Research Reactor Center, University of Missouri, Columbia, Missouri, USA; 8 Division of Epidemiology and Community Health, Cancer Center, University of Minnesota, Minneapolis, Minnesota, USA

**Keywords:** arsenic, DNA repair polymorphism, nucleotide excision repair, skin cancer

## Abstract

**Background:**

Arsenic exposure may alter the efficiency of DNA repair. UV damage is specifically repaired by nucleotide excision repair (NER), and common genetic variants in NER may increase risk for non-melanoma skin cancer (NMSC).

**Objective:**

We tested whether polymorphisms in the NER genes *XPA* (A23G) and *XPD* (Asp312Asn and Lys751Gln) modify the association between arsenic and NMSC.

**Methods:**

Incident cases of basal and squamous cell carcinoma (BCC and SCC, respectively) were identified through a network of dermatologists and pathology laboratories across New Hampshire. Population-based controls were frequency matched to cases on age and sex. Arsenic exposure was assessed in toenail clippings. The analysis included 880 cases of BCC, 666 cases of SCC, and 780 controls.

**Results:**

There was an increased BCC risk associated with high arsenic exposure among those homozygous variant for *XPA* [odds ratio (OR) = 1.8; 95% confidence interval (CI), 0.9–3.7]. For *XPD*, having variation at both loci (312Asn and 751Gln) occurred less frequently among BCC and SCC cases compared with controls (OR = 0.8; 95% CI, 0.6–1.0) for both case groups. In the stratum of subjects who have variant for both *XPD* polymorphisms, there was a 2-fold increased risk of SCC associated with elevated arsenic (OR = 2.2; 95% CI, 1.0–5.0). The test for interaction between *XPD* and arsenic in SCC was of borderline significance (*p* < 0.07, 3 degrees of freedom).

**Conclusions:**

Our findings indicate a reduced NMSC risk in relation to *XPD* Asp312Asn and Lys751Gln variants. Further, these data support the hypothesis that NER polymorphisms may modify the association between NMSC and arsenic.

Arsenic, classified as a human carcinogen, is a pervasive, naturally occurring mineral [International Agency for Research on Cancer 2004; U.S. Environmental Protection Agency (EPA) 2001]. A common route of exposure is through groundwater, where arsenic leaches in from surrounding rock. Countries with high endemic arsenic in groundwater have provided evidence of an association between arsenic and non-melanoma skin cancer (NMSC) as well as precursor skin lesions such as hyperkeratosis. For example, ecologic studies in Taiwan reported a dose–response relationship between arsenic in groundwater and prevalence of skin conditions, including NMSC (Chen et al. 1985; Hsueh et al. 1995; Tseng et al. 1968). A cohort study in this same area also observed a dose–response relationship with incidence of NMSC (Hsueh et al. 1997). Moreover, an association between NMSC and arsenic in drinking water was reported for studies in Silesia, Argentina, Mexico, and Chile (Bergoglio 1964; Cebrian et al. 1983; Guo et al. 1997; Jackson and Grainge 1975; Zaldivar 1974).

However, in the United States, arsenic concentrations in drinking water are much lower. Municipal water is regulated by the U.S. EPA, with the standard presently set at 10 μg/L (U.S. EPA 2001); however, private sources of water (defined as serving fewer than 15 households or 25 individuals) are not required to meet this standard. In New Hampshire, approximately 40% of residents have a private source of drinking water (Karagas et al. 2002). A previous analysis of NMSC and arsenic exposure in New Hampshire found an elevated risk associated with the highest concentrations of exposure (Karagas et al. 2001).

It has been hypothesized that arsenic acts as a skin carcinogen by enhancing the effects of ultraviolet (UV) radiation (Hartwig 1998; Rossman 2003). *In vitro* studies have demonstrated that arsenic inhibits the ligation (Hartwig 1998; Hartwig et al. 1997; Lee-Chen et al. 1992) and incision steps of nucleotide excision repair (NER), even at low concentrations (Hartwig 1998; Hartwig et al. 1997). Others have shown inorganic arsenic to be only weakly mutagenic, whereas DNA damage and mutation frequency after exposure to both arsenic and UV radiation is more than additive (Danaee et al. 2004). Although many biologic pathways may be disrupted by arsenic exposure, including interfering with the cell cycle activities of p53 or inhibiting base excision repair through reduced DNA ligase III or poly-(ADP-ribose)polymerase activity (Li and Rossman 1989a, 1989b, 1991; Vogt and Rossman 2001; Yager and Wiencke 1997), the most compelling candidate for NMSC among Caucasians is the NER pathway, given the specificity of NER to repair damage from exposure to UV radiation.

Epidemiologic studies have examined the relationship of polymorphisms in the NER genes *Xeroderma pigmentosum* group*s A* and *D* [*XPA* and *XPD*; Unigene accession numbers P23025, S10888, respectively (Unigene 2007)] and cancer risk. The A23G polymorphism in *XPA* (rs1800975), located four nucleotides upstream of the start codon, has been reported to influence the risk of lung cancer and NMSC (Butkiewicz et al. 2004; Miller et al. 2006; Park et al. 2002; Popanda et al. 2004; Wu et al. 2003). This polymorphism is located in the Kozak sequence, and coding changes in this region are thought to influence protein levels (Kozak 1987, 1996). In fact, having one or more copies of the wild-type G allele for this polymorphism has been reported to lead to significantly higher DNA repair capacity (DRC), as determined by the host-cell reactivation assay (Wu et al. 2003). The reduced repair phenotype has been associated with risk of NMSC and other cancers (Berwick and Vineis 2000; Wei et al. 1994). We have previously demonstrated that this single polymorphism captures risk information for the *XPA* haplotype in NMSC, and that the A allele is associated with reduced risk of both basal and squamous cell carcinomas (BCC and SCC, respectively) (Miller et al. 2006).

Polymorphisms in *XPD* have also been associated with DRC and NMSC susceptibility, with particular emphasis on two non-synonymous polymorphisms: Asp312Asn (G→A; rs1799793) polymorphism in exon 10 and Lys751Gln (A→C; rs13181) in exon 23. The wild-type alleles for these polymorphisms were found to have better DRC than the variants as determined by the host-cell reactivation assay (Spitz et al. 2001), although other studies containing fewer subjects did not observe the same relationship between these polymorphisms and DNA repair (Duell et al. 2000; Lunn et al. 2000; Moller et al. 1998). However, when these polymorphisms were examined jointly (i.e., haplotypes), alleles with multiple variants were consistently observed at greater frequency among controls than BCC or SCC cases (Han et al. 2005; Lovatt et al. 2005). However, the evidence was less strong when these polymorphisms were investigated singly (Dybdahl et al. 1999; Festa et al. 2005; Vogel et al. 2001). Results from Denmark have consistently found a nonsynonymous polymorphism at codon 156 to be associated with NMSC (Dybdahl 1999; Lovatt et al. 2005; Vogel et al. 2001, 2005).

The hypothesis that *XPD* polymorphisms interact with arsenic in skin lesions has been tested in Bangladesh, where substantially elevated groundwater levels of arsenic occur. In this previous study (Ahsan et al. 2003), the increased risk of hyperkeratosis associated with arsenic exposure was stronger among those with the Lys/Lys genotype. In the current study we have extended these observations to test whether two polymorphisms in *XPD*, and variation in *XPA*, modify the skin cancer risk associated with arsenic in a U.S. Caucasian population.

## Materials and Methods

### Study population

Cases of primary invasive SCC and BCC were identified through a collaboration with dermatologists and pathologists serving the population of New Hampshire (Karagas et al. 1999). We selected New Hampshire residents between 25 and 74 years of age who were diagnosed with SCC or BCC between 1 July 1993 and 30 June 1995 (series 1) and between 1 July 1997 and 30 March 2000 (series 2). Participants were required to have an identifiable telephone number and speak English to be eligible. We identified all potentially eligible cases of SCC and, for efficiency, randomly selected a sample of BCC cases (stratified on age, sex, and anatomic site to ensure representativeness of the entire BCC group) in ratios of approximately 2:1 in the first series and 1:1 in the second series. Potential controls less than 65 years of age were chosen from the New Hampshire Department of Transportation driver’s license files of state residents, and those 65 years and older were identified through the Center for Medicare and Medicaid Services files of New Hampshire residents enrolled in Medicare. Controls were frequency-matched to the combined BCC and SCC case distribution on sex and age (25–35, 36–45, 46–50, 51–59, 60–64, 65–69, 70–74 years). The Committee for the Protection of Human Subjects of Dartmouth College approved the study, and participants provided written informed consent according to the approved protocol. Participants were interviewed, usually in their home, to obtain information on demographic factors (e.g., ethnicity, education), sun exposures (e.g., number of severe sunburns), and UV sensitivity (e.g., tendency to tan or burn). To minimize potential reporting biases, interviewers and participants were blinded to study hypotheses, and interviewers were blinded to case–control status of participants. More detailed information on data collected in the interview is provided elsewhere (Miller et al. 2006).

### Toenail arsenic

To obtain a biomarker of ingested arsenic, toenail clippings from study participants were obtained. Arsenic was measured using instrumental neutron activation analysis at the University of Missouri Research Reactor Center. This method has been described in detail by Nichols et al. (1998). Briefly, samples first were washed to remove external contamination. Quality controls consisted of matrix-matched samples with known content and analytical blanks. The coefficient of variation between assays was 8% (Karagas et al. 2001). The detection limit for arsenic was 0.001 μg/g. All samples were blinded to case–control status.

### Genotyping

At the time of interview, blood specimens were collected for DNA extraction. In cases where it was not possible to collect a blood specimen, a buccal specimen was retrieved. To extract buccal cell DNA, mouthwash rinses were centrifuged at 3,200 rpm for 15 min to pellet buccal cells, followed by a wash of 15 mL TE (Tris–EDTA) buffer and spinning for 15–20 sec to resuspend pellet. DNA was extracted from buffy coat and buccal cell pellets with QIAamp DNA extraction kits (Qiagen, Valencia, CA). ABI Taqman chemistry was used to genotype the three NER polymorphisms on an ABI7000 (*XPA*: G→A (–4) (rs1800975); *XPD*: Asp312Asn (rs1799793) and Lys751Gln (rs13181)). Taqman primers, probes, and conditions are available upon request. For quality assurance, positive and negative controls were used in each genotyping run, and 20% of samples were imbedded duplicates. Laboratory personnel were blinded to case–control status.

### Statistical analysis

All analyses were conducted separately for BCC and SCC cases. The genotype frequency for each polymorphism was tested for Hardy-Weinberg equilibrium. Unconditional logistic regression models generated odds ratios (ORs) and 95% confidence intervals (CIs) to examine the relationship between each NER polymorphism in the two case groups compared with controls. Multivariate models controlled for age and sex, along with number of severe sunburns, and a pigment score. The pigment score was generated as a multivariate confounder score (Cook and Goldman 1989; Miettinen 1976). A higher pigment score was consistent with lighter pigmentation, which leads to higher UV exposure in keratinocytes (target cells for NMSC). As described by Miller et al. (2006), the pigment score summarized the contribution of multiple potential risk factors, including skin reaction to first hour of intense sunshine (tan only, mild burn then tan, burn), skin reaction to repeated sun exposure (deep tan, moderately tan, mild tan and peel, freckling or no tan), hair color (dark brown/black, light brown, red, blond), eye color (brown/black, green/hazel, blue/gray), skin color (medium/dark, light), and number of moles on back (0, 1, 2–4,≥ 5) into a single variable. This score was included in the models of interest as quartiles, as determined by the distribution in controls.

We modeled the main effects of the NER polymorphisms with genotypes dichotomized as homozygous wild-type compared with heterozygous and homozygous variant. Based on prior work in which we observed an elevated risk at the upper end of the exposure distribution (Karagas et al. 2002), we dichotomized exposure at ≤0.286 μg/g to examine gene–environment interactions. The joint effects between the polymorphisms and arsenic were modeled, using the homozygous wild-type low arsenic (≤0.286 μg/g) as the reference category and the ORs and 95% CIs for the remaining genotype–arsenic categories were estimated. To test for statistical interaction, logistic models included separate terms for arsenic and genotype and also contained their cross products. The likelihood ratio tests were conducted leaving off the cross products and comparing the –2 log likelihoods from these models. Further, it has been suggested that arsenic-induced NMSC may be more likely to develop on low-UV exposure regions of the skin (Castren et al. 1998; Tapio and Grosche 2006). To examine this in our data, tumors were determined to occur on a high UV-exposure site (e.g., head or neck) or a sun-protected site. However, our results did not differ by tumor location (data not shown). Statistics were generated using SAS (version 9.1; SAS Institute Inc., Cary, NC). All tests were two-sided, and a *p*-value of 0.05 was considered statistically significant.

A total of 1,181 BCC, 833 SCC, and 1,066 controls participated in the study (participation in series 1: 82% of cases and 69% of controls; participation in series 2: 81% of cases and 76% of controls). Subjects provided DNA samples from blood and/or buccal specimens (86% of series 1 and 85% of series 2 subjects). Ninety-eight percent of subjects provided toenail samples. Because the etiology for NMSC may vary by race and because of the low numbers of non-Caucasians identi-fied, we restricted this analysis to Caucasians. Only Caucasians with both genotyping and toenail arsenic data available were included in this analysis (780 controls, 880 BCC, and 666 SCC).

## Results

The majority of BCC and SCC cases were male and overall were approximately 60 years of age. After adjusting for age and sex, the number of severe sunburns was significantly higher among both BCC (*p* < 0.01) and SCC (*p* < 0.01) cases than controls, and pigment score also was higher among NMSC case groups than controls (BCC, *p* < 0.01; SCC, *p* < 0.01) (Table 1). There were no meaningful differences in the distribution of these characteristics for subjects who provided DNA and toenail specimens compared with those who did not (data not shown).

For all three polymorphisms, the distribution of the genotypes in controls was in Hardy-Weinberg equilibrium. The main effect models for NER genotypes are presented in Table 2. In the *XPA* A23G polymorphism model, BCC and SCC cases were less likely than controls to carry one or two copies of the variant A allele (BCC: OR = 0.8, 95% CI, 0.7–1.0; SCC: OR = 0.8, 95% CI, 0.7–1.0), and these differences were of borderline significance, as described previously (Miller et al. 2006). Similarly, for the Asp312Asn polymorphism in *XPD*, genotypes containing the variant allele were less frequent in both case groups, and this was of borderline significance (BCC: OR = 0.8, 95% CI, 0.7–1.0; SCC: OR = 0.8, 95% CI, 0.6–1.0). However, the Lys751Gln in this same gene suggested no association with either BCC or SCC (BCC: OR = 0.9, 95% CI, 0.7–1.1; SCC: OR = 0.9, 95% CI, 0.7–1.1).

Estimates of the joint effects of genotypes and arsenic are provided in Table 3. For *XPA*, subjects with homozygous wild-type genotypes and high arsenic (> 0.286 μg/g) had an elevated risk for BCC compared with the homozygous wild-type with lower arsenic (*XPA*: OR = 1.8; 95% CI, 0.9–3.7), although the test for interaction was not statistically significant (*p* = 0.24). The test for interaction suggested that the two polymorphisms in *XPD* were the stronger modifiers of the association between arsenic and NMSC; the Asp312Asn polymorphism–arsenic interaction was of borderline significance for SCC (*p* = 0.08), and the Lys751Gln polymorphism–arsenic interaction was statistically significant for SCC (*p* = 0.03). For these two polymorphisms, the risk of SCC was stronger among carriers of the variant with high arsenic compared with the homozygous wild-type with high arsenic.

We examined the degree of linkage disequilibrium between these *XPD* polymorphisms among controls and found Asp312Asn and Lys751Gln did co-segregate (*D*′ = 0.68). Therefore, we generated logistic models to examine how combinations of alleles at these polymorphisms influenced the risk of NMSC (Table 4). The lower frequency of the variant alleles among the BCC and SCC cases relative to contols reported earlier for each polymorphism, only existed when there was at least one variant allele at both codons 312 and 751 (BCC: OR = 0.8, 95% CI, 0.6–1.0; SCC: OR = 0.8, 95% CI, 0.6–1.0). Examining gene–environment interaction with the combined *XPD* genotype revealed a borderline significant interaction for SCC (Table 5, *p* for interaction = 0.07, 3 df). Among subjects with one or more variants at both of the *XPD* polymorphisms, there was a 2-fold risk of SCC (OR = 2.2; 95% CI, 1.0–5.0) or those with high arsenic (> 0.286 μg/g) relative to those with lower arsenic.

## Discussion

Previous analyses of this population have found arsenic was a potential risk factor for NMSC in the United States (Karagas et al. 2001, 2002), and the current analysis suggests that the relationship between arsenic and NMSC may be modified by NER polymorphisms. Having at least one variant at both *XPD* polymorphisms was observed less frequently in both BCC and SCC cases than in controls. Among these carriers of variants at multiloci, subjects with toenail arsenic > 0.286 μg/g had twice the risk of SCC compared with those with lowest arsenic exposure. The results from the *XPA* analysis suggested that an elevated risk of BCC from exposure to high arsenic may occur in subjects homozygous wild-type for the A23G polymorphism.

Previous studies have examined the *XPD* codon 751 polymorphism in relation to benign keratoses. In a study of residents of Bangladesh, Ahsan et al. (2003) examined the relationship between urinary arsenic, *XPD* Lys751Gln, and risk of hyperkeratosis, a potential precursor lesion for NMSC. The investigators found a statistically significant trend for increased arsenic-associated hyper-keratosis among those with the Lys/Lys genotype and a weaker dose response for subjects with one or two copies of the *XPD* codon 751 variant allele. In West Bengal India, investigators reported suboptimal DNA repair (as measured by frequency of chromosomal aberrations and aberrant cells) among arsenic-exposed subjects with the Lys/Lys genotype compared with those with one or more variant alleles (Banerjee et al. 2007). Those with the Lys/Lys genotype also were at greater risk of hyperkeratosis. However, there are several key differences between the studies in Asia and ours from the United States. First, the minor allele frequency was higher in Bangladesh (50%) and India (40%) than in U.S. Caucasians (30%) (Ahsan et al. 2003; Banerjee et al. 2007; Shen et al. 1998; Spitz et al. 2001). Pigmentary differences influence the amount of UV radiation exposure that reaches keratinocytes, which may alter the nature of the arsenic–gene interaction. In addition, the differences in arsenic dose may underlie the population differences in gene–environment interaction, as studies have shown that the reaction of cells to arsenic exposure differs by dose (Andrew et al. 2006; Barchowsky et al. 1999; Del Razo et al. 2001). These differences in dose are dramatic; arsenic in drinking water in Bangladesh ranges from 10 to 2,040 μg/L (Tondel et al. 1999), whereas in New Hampshire the range is considerably lower (0.01–180 μg/L among controls) (Karagas et al. 1998). Finally, although hyperkeratosis may be a precursor lesion for some types of keratinocyte malignancies, in particular SCC, it is not a malignant end point. Our study included incident, histologically confirmed invasive squamous cell carcinomas.

There are a number of ways that arsenic may interfere with the NER pathway and removal of DNA lesions, such as pyrimidine dimers and 6,4-photoproducts from UV radiation (Danaee et al. 2004; Hartwig 1998, 1997; Lee-Chen et al. 1992; Rossman 2003). First, NER proteins have zinc fingers, where zinc is surrounded by four cysteine and/or histidine residues containing sulfhydryl groups (Mackay and Crossley 1998). Arsenic has a high affinity for sulfhydryl groups, which would allow arsenic to bind to the repair proteins. Consequently, this would inhibit the ability of NER proteins to repair DNA damage, and as a result, increase the risk of cancer. The 312 and 751 polymorphisms in *XPD* alter the acidity of the amino acids on the protein. If these non-synonymous polymorphisms change protein structure, this could influence arsenic–protein binding, which may explain the mechanism of *XPD* polymorphism effect modification.

Another hypothesis involves the expression of NER genes. Arsenic has been demonstrated to reduce the expression of NER genes, including the incision proteins ERCC1 and XPF (Andrew et al. 2003). This reduced expression could decrease DRC, a phenotype which has been linked to cancer susceptibility, including skin cancer (Brockmoller et al. 2000; Wei et al. 1994). The polymorphisms we studied in *XPA* and *XPD* have already been linked to influencing DRC (Hemminki et al. 2001; Qiao et al. 2002a; Spitz et al. 2001; Wu et al. 2003). If arsenic influenced gene expression levels and therefore DRC, the polymorphisms may further amplify the association between arsenic and NMSC.

We observed that, among those not exposed to high arsenic, subjects with genotypes containing the variant allele were at reduced risk for NMSC. This is consistent with other studies of NMSC and polymorphisms in DNA repair genes (Han et al. 2005, 2004; Marin et al. 2004; Nelson et al. 2005; Popanda et al. 2004; Sanyal et al. 2004; Shen et al. 2001). Keratinocytes are thought to have a greater apoptotic response to DNA damage than other cell types (Bowen et al. 2003). As a result, keratinocytes containing the variant allele, which may have suboptimal DNA repair compared with the wild-type (described earlier), are more likely to undergo apoptosis due to insufficient repair of DNA damage, which can reduce the risk of NMSC (Nelson et al. 2002). However, when subjects are exposed to higher concentrations of arsenic, there may be interference with cell machinery that influences DNA damage burden and the apoptotic threshold. As a result, cells with the variant allele may have more DNA damage that is not repaired and be unable to undergo apoptosis, resulting in an increased risk of NMSC.

We chose to examine the relationship between arsenic, NER, and NMSC with these particular polymorphisms because previous studies found that they influenced susceptibility to various cancers. However, there are many other polymorphisms in *XPA* and *XPD*, which raises the question of whether other polymorphisms could influence these associations and should be analyzed simultaneously. For *XPA*, we previously conducted a haplotype analysis, accounting for coding throughout the gene (Miller et al. 2006). These results suggested that the association with NMSC susceptibility was captured by the A23G polymorphism and that haplotypes accounting for variation across *XPA* did not contribute more information. Therefore, we focused on the A23G polymorphism for this gene. For *XPD*, we chose to focus on two nonsynonous polymorphisms. We observed that the 312 and 751 polymorphisms in *XPD* were in linkage disequilibrium, which has been reported by other investigators (Butkiewicz et al. 2001; Caggana et al. 2001; Han et al. 2005; Hou et al. 2002; Qiao et al. 2002b; Spitz et al. 2001; Vogel et al. 2001). In addition, the combined genotype data for the two polymorphisms suggested that having coding changes at both loci together influenced risk of NMSC more than just one coding change. Therefore, our results suggest that these polymorphisms should be considered together and may identify individuals who are more susceptible to the carcinogenicity of arsenic. At this point we do not know how additional coding variation in *XPD* would influence this finding.

By using toenail measurements of arsenic, we have a measure of arsenic intake through all routes of exposure. A limitation of this measure is that it reflects exposure at one point in time. As previously reported, this New Hampshire population was relatively stable, with over half of subjects using the same water system for at least 15 years (Karagas et al. 2001). In our study population, arsenic in toenails measured 3–5 years apart were correlated (Karagas et al. 2001), and in the Nurses’ Health Study measurements 6 years apart were correlated (Garland et al. 1993). In addition, because our arsenic measurements were blinded to case status, exposure misclassification would result in attenuated estimates.

Our findings provide additional support for the co-carcinogenic action of arsenic via the NER pathway. Additional work is needed to further define the biologic mechanism underlying the interaction, as well as confirm these results in a second population. We chose to focus on a particular mechanism of arsenic co-carcinogenicity with UV radiation in order to identify individuals who may be most susceptible to the effects of arsenic. More research is needed to determine how chronic exposure to low concentrations of arsenic in groundwater as experienced in the United States contributes to the risk of NMSC.

## Figures and Tables

**Table 1:** Selected characteristics of basal cell carcinoma (*n* = 880) and squamous cell carcinoma (*n* = 666) cases and controls (*n* = 780) on whom toenail arsenic and genotype data were available.

Characteristic	Controls [*n* (%)]	BCC [*n* (%)]	SCC [*n* (%)]
Sex			
Male	478 (61.3)	498 (56.6)	423 (63.5)
Female	302 (38.7)	382 (43.4)	243 (36.5)
Mean age (years ± SD)	61.2 ± 10.5	58.8 ± 11.1	64.1 ± 8.7
Severe sunburns^a^			
0–2	471 (61.3)	402 (46.2)	311 (47.4)
≥3	297 (38.7)	469 (53.8)	345 (52.6)
Pigment score^b^,^c^			
Quartile 1	195 (25.0)	83 (9.4)	66 (9.9)
Quartile 2	197 (25.3)	173 (19.7)	114 (17.1)
Quartile 3	198 (25.4)	244 (27.7)	179 (26.9)
Quartile 4	190 (24.4)	380 (43.2)	307 (46.1)

a Missing sunburn information: 12 controls, 9 BCC, 10 SCC.

b Pigment score was generated using a multivariate confounder score and combined data on the following pigment factors: skin reaction to first hour of intense sunshine; skin reaction to repeated sun exposure, hair color, eye color, skin color, and number of moles on back; higher pigment score represented lower pigmentation and melanin production.

c The distribution of pigment score in controls represents the average for the pigment score in controls as generated separately for BCC and SCC.

**Table 2:** Association between nucleotide excision repair polymorphisms and NMSC.

Gene, polymorphism	Controls [*n* (%)]	BCC [*n* (%)]	OR (95% CI)^a^	SCC [*n* (%)]	OR (95% CI)^a^
*XPA*, A23G	*n* = 773	*n* = 868		*n* = 662	
GG	347 (44.9)	428 (49.3)	Referent	322 (48.6)	Referent
AG, AA	426 (55.1)	440 (50.7)	0.8 (0.7–1.0)	340 (51.4)	0.8 (0.7–1.0)
A allele frequency (%)	34.1	30.8		31.1	
*XPD*, Asp312Asn	*n* = 728	*n* = 782		*n* = 631	
Asp/Asp	301 (41.4)	347 (44.4)	Referent	286 (45.3)	Referent
Asp/Asn, Asn/Asn	427 (58.7)	435 (55.6)	0.8 (0.7–1.0)	345 (54.7)	0.8 (0.6–1.0)
Asn frequency (%)	35.1	33.8		32.9	
*XPD*, Lys751Gln	*n* = 753	*n* = 854		*n* = 644	
Lys/Lys	322 (42.8)	395 (46.3)	Referent	289 (44.9)	Referent
Lys/Gln, Gln/Gln	431 (57.2)	459 (53.8)	0.9 (0.7–1.1)	355 (55.1)	0.9 (0.7–1.1)
Gln frequency (%)	34.8	33.3		33.4	

a Odds ratios controlled for age, sex, severe sunburns, and pigmentation.

**Table 3:** Joint effects of toenail arsenic measurements and nucleotide excision repair polymorphisms on risk of NMSC.

Gene, polymorphism	Toenail arsenic (μg/g)	Controls [*n* (%)]	BCC [*n* (%)]	OR (95% CI)^a^	SCC [*n* (%)]	OR (95% CI)^a^
*XPA*, A23G		*n* = 773	*n* = 868		*n* = 662	
GG	≤ 0.286	334 (43.2)	399 (46.0)	Referent	309 (46.7)	Referent
	> 0.286	13 (1.7)	29 (3.3)	1.8 (0.9–3.7)	13 (2.0)	1.1 (0.5–2.6)
AG, AA	≤ 0.286	401 (51.9)	413 (47.6)	0.8 (0.7–1.0)	323 (48.8)	0.8 (0.7–1.1)
	> 0.286	25 (3.2)	27 (3.1)	0.9 (0.5–1.6)	17 (2.6)	0.8 (0.4–1.6)
*p*-Value for interaction^b^				0.24		0.79
*XPD*, Asp312Asn		*n* = 728	*n* = 782		*n* = 631	
Asp/Asp	≤ 0.286	282 (38.7)	320 (40.9)	Referent	276 (43.7)	Referent
	> 0.286	19 (2.6)	27 (3.4)	1.3 (0.7–2.4)	10 (1.6)	0.6 (0.3–1.3)
Asp/Asn, Asn/Asn	≤ 0.286	409 (56.2)	411 (52.6)	0.8 (0.7–1.0)	326 (51.7)	0.8 (0.6–1.0)
	> 0.286	18 (2.5)	24 (3.1)	1.2 (0.6–2.2)	19 (3.0)	1.2 (0.6–2.4)
*p*-Value for interaction^b^				0.82		0.08
*XPD*, Lys751Gln		*n* = 753	*n* = 854		*n* = 644	
Lys/Lys	≤ 0.286	303 (40.2)	368 (43.1)	Referent	280 (43.5)	Referent
	> 0.286	19 (2.5)	27 (3.2)	1.2 (0.6–2.2)	9 (1.4)	0.6 (0.2–1.3)
Lys/Gln, Gln/Gln	≤ 0.286	416 (55.3)	431 (50.5)	0.8 (0.7–1.0)	336 (52.2)	0.8 (0.6–1.0)
	> 0.286	15 (2.0)	28 (3.3)	1.6 (0.8–3.2)	19 (2.9)	1.6 (0.8–3.4)
p-Value for interaction^b^				0.31		0.03

a Odds ratios controlled for age, sex, severe sunburns, and pigmentation.

b From test of interaction with 1 df.

**Table 4:** Combination of *XPD* polymorphisms Asp312Asn and Lys751Gln and risk of NMSC in BCC (*n* = 771), and SCC (*n* = 617), and controls (*n* = 710).

*XPD* 312	*XPD* 751	Controls[*n* (%)]	BCC [*n* (%)]	OR (95% CI)^a^	SCC [*n* (%)]	OR (95% CI)^a^
Asp/Asp	Lys/Lys	236 (33.2)	271 (35.2)	Referent	221 (35.8)	Referent
	Lys/Gln, Gln/Gln	60 (8.5)	72 (9.3)	1.0 (0.7–1.6)	60 (9.7)	1.1 (0.7–1.6)
Asp/Asn, Asn/Asn	Lys/Lys	55 (7.8)	79 (10.3)	1.1 (0.7–1.7)	53 (8.6)	1.0 (0.6–1.6)
	Lys/Gln, Gln/Gln	359 (50.6)	349 (45.3)	0.8 (0.6–1.0)	283 (45.9)	0.8 (0.6–1.0)

a Odds ratios controlled for age, sex, severe sunburns, and pigmentation.

**Table 5:** *XPD* polymorphisms Asp312Asn and Lys751Gln, toenail arsenic, and risk of NMSC in BCC (*n* = 771), SCC (*n* = 617), and controls (*n* = 710).

Toenail arsenic (μg/g)	*XPD* 312	*XPD* 751	Controls [*n* (%)]	BCC [*n* (%)]	OR (95% CI)^a^	SCC [*n* (%)]	OR (95% CI)^a^
≤ 0.286	Asp/Asp	Lys/Lys	219 (30.9)	249 (32.3)	Referent	214 (34.7)	Referent
		Lys/Gln, Gln/Gln	58 (8.2)	67 (8.7)	1.0 (0.7–1.5)	57 (9.2)	1.0 (0.6–1.5)
	Asp/Asn, Asn/Asn	Lys/Lys	53 (7.5)	77 (10.0)	1.1 (0.7–1.7)	52 (8.4)	1.0 (0.6–1.5)
		Lys/Gln, Gln/Gln	347 (48.9)	328 (42.5)	0.8 (0.6–1.0)	267 (43.3)	0.7 (0.6–0.9)
> 0.286	Asp/Asp	Lys/Lys	17 (2.4)	22 (2.9)	1.1 (0.6–2.2)	7 (1.1)	0.5 (0.2–1.2)
		Lys/Gln, Gln/Gln	2 (0.3)	5 (0.7)	2.6 (0.5–14.7)	3 (0.5)	1.4 (0.2–8.9)
	Asp/Asn, Asn/Asn	Lys/Lys	2 (0.3)	2 (0.)	1.1 (0.1–7.7)	1 (0.2)	0.4 (0.03–6.2)
		Lys/Gln, Gln/Gln	12 (1.7)	21 (2.7)	1.6 (0.7–3.4)	16 (2.6)	1.7 (0.7–3.8)
*p*-Value for interaction^b^					0.61		0.07

a Odds ratios controlled for age, sex, severe sunburns, and pigmentation.

b From test for interaction (3 df) between arsenic and the combined *XPD* polymorphisms at codons 312 and 751.
